# Automated detection of altered mental status in emergency department clinical notes: a deep learning approach

**DOI:** 10.1186/s12911-019-0894-9

**Published:** 2019-08-19

**Authors:** Jihad S. Obeid, Erin R. Weeda, Andrew J. Matuskowitz, Kevin Gagnon, Tami Crawford, Christine M. Carr, Lewis J. Frey

**Affiliations:** 1 Biomedical Informatics Center, Medical University of South Carolina, Charleston, SC USA; 2 Department of Public Health Sciences, Medical University of South Carolina, Charleston, SC USA; 3 Department of Clinical Pharmacy and Outcome Sciences, Medical University of South Carolina, Charleston, SC USA; 4 Department of Emergency Medicine, Medical University of South Carolina, Charleston, SC USA; 5 Department of Computer Science and Engineering, University of South Carolina, Columbia, SC USA

**Keywords:** Altered mental status, Machine learning, Deep learning, Word embedding, Pulmonary embolism, Decision support

## Abstract

**Background:**

Machine learning has been used extensively in clinical text classification tasks. Deep learning approaches using word embeddings have been recently gaining momentum in biomedical applications. In an effort to automate the identification of altered mental status (AMS) in emergency department provider notes for the purpose of decision support, we compare the performance of classic bag-of-words-based machine learning classifiers and novel deep learning approaches.

**Methods:**

We used a case-control study design to extract an adequate number of clinical notes with AMS and non-AMS based on ICD codes. The notes were parsed to extract the history of present illness, which was used as the clinical text for the classifiers. The notes were manually labeled by clinicians. As a baseline for comparison, we tested several traditional bag-of-words based classifiers. We then tested several deep learning models using a convolutional neural network architecture with three different types of word embeddings, a pre-trained word2vec model and two models without pre-training but with different word embedding dimensions.

**Results:**

We evaluated the models on 1130 labeled notes from the emergency department. The deep learning models had the best overall performance with an area under the ROC curve of 98.5% and an accuracy of 94.5%. Pre-training word embeddings on the unlabeled corpus reduced training iterations and had performance that was statistically no different than the other deep learning models.

**Conclusion:**

This supervised deep learning approach performs exceedingly well for the detection of AMS symptoms in clinical text in our environment. Further work is needed for the generalizability of these findings, including evaluation of these models in other types of clinical notes and other environments. The results seem promising for the ultimate use of these types of classifiers in combination with other information derived from the electronic health records as input for clinical decision support.

**Electronic supplementary material:**

The online version of this article (10.1186/s12911-019-0894-9) contains supplementary material, which is available to authorized users.

## Background

The use of electronic health records (EHR) to identify specific clinical phenotypes has gained significant momentum over the past several years at both the local and national stages [1–3]. A good portion of the information within the EHR resides in free-text format contained inside numerous types of clinical notes [2, 4]. Characterizing patients based on EHR has several useful purposes including: identification of participants for research recruitment [5, 6], population health and epidemiological studies [7–9] and clinical decision support [4, 10–12].

### The clinical use case

This study was motivated by the need for the assessment of mental status during the evaluation and risk stratification of patients with pulmonary embolism in the emergency department (ED) [13, 14]. Our objective is to automate the detection of altered mental status (AMS) in ED provider notes for the ultimate use in clinical decision support. Pulmonary embolism should be considered during the evaluation of patients with syncope [15, 16]. The Pulmonary Embolism Severity Index (PESI) is a risk stratification guideline that helps clinicians assess patients with pulmonary embolism [13, 14] and determine the necessary practice guidelines for treatment and follow-up care. According to the PESI guideline, the presence of AMS significantly increases the risk of post-pulmonary embolism mortality. Based on these reports, and for the purpose of this experiment, we defined AMS as the presence of any of the following symptoms: disorientation, confusion, somnolence, lethargy, stupor, syncope or coma. Most of the other patient characteristics needed for PESI can be extracted from coded EHR data, e.g. age, sex, and vital signs among others [14]. However, the presence or absence of AMS requires extraction of the information from the providers’ clinical text notes. Although International Classification of Diseases (ICD) codes are commonly used for phenotyping patients based on EHR [9, 17, 18], coder errors, such as misattribution, unbundling, and upcoding, result in low sensitivity and specificity for retrieval of reliable clinical information [19, 20].

### NLP and machine learning approaches

Several natural language processing (NLP) pipelines have been reported in recent years that utilize combinations of essential components including: tokenization, part-of-speech tagging, named entity recognition, negation and mapping to Unified Medical Language System (UMLS) ontologies [21–23]. These pipelines utilize a variety of machine learning algorithms to accomplish certain tasks including named entity recognition. Machine learning has also been used extensively in clinical text classification tasks. Notable examples include: the detection of influenza in ED notes [24], the identification of hepatobiliary disease and acute renal failure in general clinical notes [25], and the identification of child abuse cases in a large set of text notes in a public health organization in the Netherlands [26]. The authors in these studies have used popular classifiers such as Naïve Bayes Classifier [27], Support Vector Machine [28] and Random Forest [29]. Although deep learning approaches [30], for example convolutional neural networks have been used in predictive modeling in the clinical domain, there is a limited amount of literature on deep learning applications for clinical text classification that is focused on detecting specific signs or symptoms as is needed for our clinical use case [31]. A recent example of a deep learning application is presented by Rajkomar et al., in which the authors describe using both structured and unstructured data for the prediction of patient readmissions [32]. In the non-clinical domain deep learning approaches have been used extensively in text classification in such tasks as sentiment analysis and movie reviews [33]. A good amount of neural network research in the area of text processing has involved unsupervised learning word vector representations or word embeddings such as word2vec [34] in an effort to derive semantic context of words. These learned word vectors could in turn be used for clinical text classification tasks [35, 36]. Pre-training models using this method provides syntactic and semantic word similarities expressed in a multi-dimensional vector space with the potential for improving classifications based on neural networks with lower computational cost [34].

In this study we evaluate the performance of several text classifiers on a simple text classification task to identify AMS in clinical notes. We compare the performance of word embedding-based deep learning models to several traditional models as a baseline, which use normalized bag-of-words representations as features. Additionally, we evaluate the impact of pre-training using word2vec on word embedding-based models. We also compare all the models to another baseline, namely identification of clinical notes with AMS using ICD codes.

## Methods

This study was approved by the Institutional Review Board for Human Research (IRB) at the Medical University of South Carolina (MUSC) under protocol number Pro00080055. We extracted provider notes for adult patients who were seen in the ED over a period of approximately 6 years, from 2012 to 2018. The notes were extracted from the Epic© EHR system [37] via the MUSC research data warehouse (RDW), which serves as an EHR data repository for research purposes. Researchers may request data from the RDW with appropriate approval and oversight.

### Patient population and clinical text corpus

We used a case-control study design to ensure an adequate balance between AMS patient records and non-AMS records (2000 patients in each group). This was done by extracting records with visits that were tagged with ICD codes indicating AMS (e.g. ICD-9 codes 799.5x and ICD-10 codes R41.x, which represent symptoms and signs involving cognition). An equal number of records were selected randomly as controls or negative cases from patients without the above specified AMS ICD codes. In order to ensure that the model is exposed to patients with thromboembolic conditions, both patient groups were enriched with patients with ICD codes for venous thromboembolism (ICD-9453.x, ICD-10 I82.x) and/or pulmonary embolism (ICD-9415.1x, ICD-10 I26.x). This was accomplished by including all patients that match these conditions within 60 days of the ED visit in the AMS group, which made up about 5% of that population as well as a balanced proportion in the non-AMS group. Based on these inclusion criteria, we received a total of 9035 raw ED provider text notes. The notes were parsed into the different sections of the clinical record using section header pattern searches to segment the text, for example, history of present illness (HPI), past medical history, physical exam, assessment, etc. However, many of the notes were incomplete or malformed and were not parsable into the respective sections. Out of the 9035, we were able to create a corpus with 8194 clinical notes belonging to 3881 patients, which were successfully parsed and included HPI and physical exam components.

### Labeling process

The parsed notes were imported into REDCap [38] and made available for review and labeling by the clinical experts on our research team, which includes two ED physicians, a clinical pharmacist and a pediatrician. REDCap (Research Electronic Data Capture) is an online data entry system widely used at academic institutions, which allows users to create data entry forms and manage the data in a secure environment [38]. The clinicians were asked to label the HPI notes as either AMS or not AMS based on a written definition that was provided on the REDCap labeling form. Altered mental status was defined as history of disorientation, lethargy, stupor, somnolence, confusion, coma, loss of consciousness, or syncope, as a component of the presenting illness. Due to time constraints, each clinician was asked to label around 250 or more of pre-assigned non-overlapping sets of notes out of the 8194, with the aim of achieving around 1000 labeled records. The labelers were also asked to drop repetitive notes for patients with frequent ED visits in order to maximize the diversity of notes, as well as notes resulting from ED visits due to severe trauma with associated loss of consciousness or due to apparent substance use since they did not fit the context of pulmonary embolism. In the end, 1130 notes out of the 8194 were labeled by the clinical team. A few cases were reviewed by more than one individual if they were deemed uncertain or not clear cut AMS or not AMS. In those cases, the notes were labeled by consensus by two or more clinicians. A sub-sample of 100 notes from the 1130 was labeled independently by two labelers in order to estimate the inter-rater reliability. Given the strict guidelines during the labeling process there was a fairly high inter-rater agreement (Cohen’s Kappa = 0.94). Table 1 shows the breakdown of the labeled notes and associated patient counts. As expected, some patients had more than one note. We had 1130 labeled notes for 858 patients.

**Table 1:** Breakdown of the labeled notes

HPI label	Notes	Patients
AMS	493	459
Not AMS	637	422
Total	1130	858^a^

^a^23 patients had records in both categories from different ED visits

In this analysis we focused on the HPI only, in order to focus on the temporal window of presentation of a patient prior to decision making by clinicians. In the context of decision support, the predicted result could then be consumed by a clinical decision support system in a timely manner. After labeling, the data was imported into R version 3.5.1 [39] for analysis.

### ICD codes

In order to assess the accuracy of ICD codes, we used the labeled data as ground truth. Table 2 shows a detailed list of the ICD codes considered to be positive for AMS in the context of risk assessment during the evaluation of patients with pulmonary embolism [14, 16]. We examined the assignment of these AMS ICD codes for encounters concurrent with the HPI notes of the ED visits. The presence of one or more of these codes during an ED visit was considered ICD positive for AMS. We report on the accuracy of ICD codes in the results section.

**Table 2:** List of ICD-9 and ICD-10 codes considered to be evidence of AMS in the context of pulmonary embolism

Code Set	ICD Code	Diagnosis Name
ICD9	780.0x	Alteration of consciousness
ICD9	780.2	Syncope and collapse
ICD9	780.97	Altered mental status
ICD9	799.5x	Signs and symptoms involving cognition
ICD10	R40.x	Somnolence, stupor and coma
ICD10	R41.0	Disorientation, unspecified
ICD10	R41.8x	Other symptoms and signs involving cognitive functions and awareness
ICD10	R41.9	Unspecified symptoms and signs involving cognitive functions and awareness
ICD10	R55	Syncope and collapse

### Text processing

In assessing all the machine learning approaches, we performed the necessary pre-processing of text for both the deep learning-based classifiers using word embeddings (WE) and the traditional bag-of-words (BOW) based models. The BOW models were used as a baseline for comparison with the deep learning models. We used the quanteda R package [40] and regular expression functions within R for the text processing pipeline. For the traditional BOW models, text processing included lower casing, removal of punctuation and stop-words, word stemming, and tokenization. For the WE models, text processing included lower casing, sentence segmentation, punctuation removal, and tokenization.

### BOW-based classifiers

In the BOW word frequencies were used as features and were normalized using term frequency–inverse document frequency (tf-idf) [41]. This resulted in a 904 (80% of 1130 used for training/cross-validation, see below) by 4765 sparse matrix for the training data, i.e. a vocabulary size of 4765 after lower casing, removal of punctuation and stop-words, and word stemming. The traditional text classification models including: Naïve Bayes Classifier (NBC) [42]; Lasso (LASS) [43]; Single Decision Tree (SDT) classifier [44] with a maximum depth of 20; Random Forest (RF) [45] with 201 trees and the number of variables randomly sampled as candidates at each split (mtry) = 150; Support Vector Machines (SVM) [46] Type 1 with a radial basis kernel, previously used successfully in text classification [47]; and an artificial neural network, with a simple multilayer perceptron (MLP) architecture, with a 64-node input layer, a 64-node hidden layer and a single output node. We used rectified linear unit (ReLU) activation function in both the input and hidden layers, and sigmoid activation for the binary output node. The MLP was trained using a learning rate of 1 × 10^− 4^, a batch size of 32, and a 10% validation split over 30 epochs.

### Word embeddings

We used Keras [48] and TensorFlow version 1.12 [49] for constructing and training the deep learning models, including the word embeddings. In preparation for word embedding, the HPI documents were converted to token sequences. In order to construct the features for the deep learning models, the sequences were padded with zeros (using pre-padding) to match the length of the longest document. We used word2vec (W2V) to generate a pre-trained model [34]. The W2V weights were derived by pre-training a W2V skip-gram model on all 8194 HPI notes (both labeled and unlabeled, i.e. unsupervised learning) using 200 dimensions per word, a skip window size of 5 words in each direction, and negative sampling of 1. In order to explore and visualize the outcome of the pre-trained W2V model, we used the t-distributed Stochastic Neighbor Embedding (t-SNE) to map the multidimensional word vectors into a 2-dimensional space [50].

### Deep learning model

**Fig. 1:**
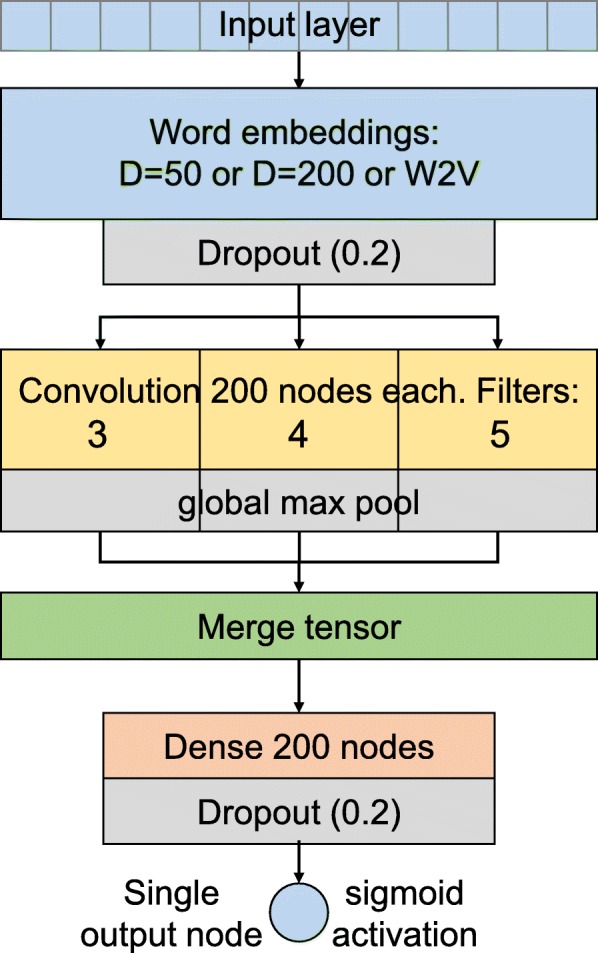
The deep neural network architecture consists of a word embedding layer, followed by a convolutional layer with multiple filters, followed by a merge tensor, a fully connected dense layer and a single sigmoid output node

We used a convolutional neural network (CNN) architecture similar to that described by Kim [33]; however, instead of using parallel channels for the embedding layer, we used either a pre-trained layer using W2V or word embedding without pre-training with either of 50 (D50) or 200 (D200) dimensions per word (Fig. 1). The input layer had a dimension size of 717, which is the size of the longest sequence of tokens + 1. The embedding layer included a drop rate of 0.2. The next layer was a convolutional layer with multiple filter sizes (3, 4 and 5) in parallel, 200 nodes each, a stride of one, and global maxpooling. This was followed by a merge tensor then a fully connected 200 node hidden layer with a drop rate of 0.2, and finally a single activation output node. We used ReLU activation function in all the layers, except for the binary output layer in which we used sigmoid activation.

Other deep neural network architectures including variations of recurrent neural networks were also tested; however, we chose the above architecture due to its superior performance and speed [33]. The CNN models were trained using an Adaptive Moment Estimation (Adam) gradient descent algorithm [51] with a learning rate = 4 × 10^− 4^, batch size = 64, validation split at 10%, and early stopping based on the loss function for the validation data with patience = 10. The early stopping allowed us to measure the number of epochs it took for each deep learning model to converge on a minimum.

### Training and evaluation

All the models were trained and evaluated using 5-fold train/test cycles using the caret package [52]. Therefore, in each cycle 20% of the data was held out (as unseen data during training) and used for testing. The area under the receiver operating characteristic (ROC) curve (AUC) along with 95% confidence intervals, and accuracies, were calculated from the combined pooled predictions of the holdout test sets collected during each of the 5-fold runs.

## Results

### ICD code analysis

Using the clinician-labeled notes as ground truth, the accuracy of identifying AMS based on the ICD codes as depicted in Table 2 and attributed to visits concurrent with the labeled HPI notes was 81.3%. Table 3 shows the distribution ICD codes for AMS over the positive and negative labels by clinicians (Cohen’s Kappa = 0.63).

**Table 3:** The confusion matrix for AMS ICD codes attributed to visits concurrent with the HPI notes vs. labels by clinicians (accuracy = 81.3%)

Label by clinician	AMS ICD’s	No AMS ICD’s
AMS	456	37
Not AMS	174	463

### BOW-based classifiers

**Fig. 2:**
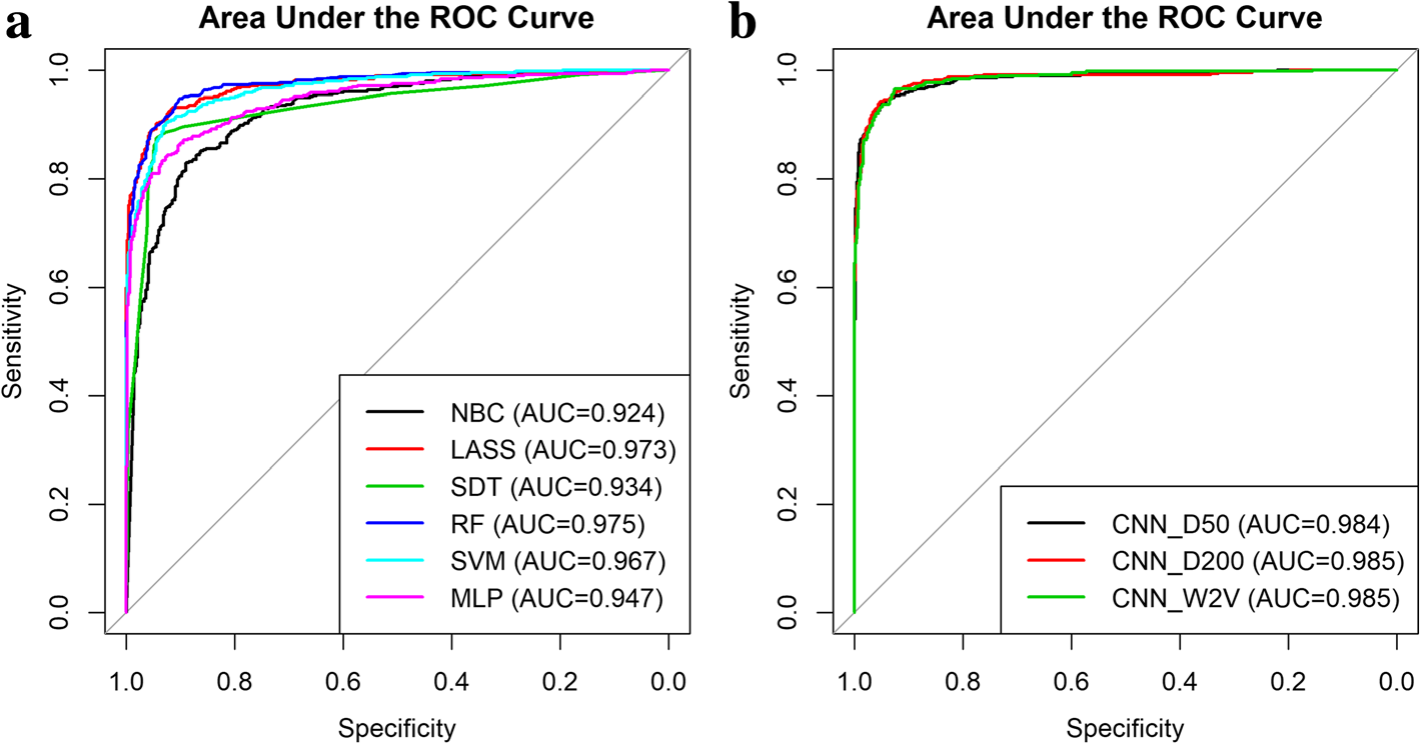
Area under the ROC curve (AUC) plots. **a**) AUC plots for the BOW-based models; **b**) AUC plots for the word embedding-based deep learning models. (Model abbreviations are described in the text)

**Fig. 3:**
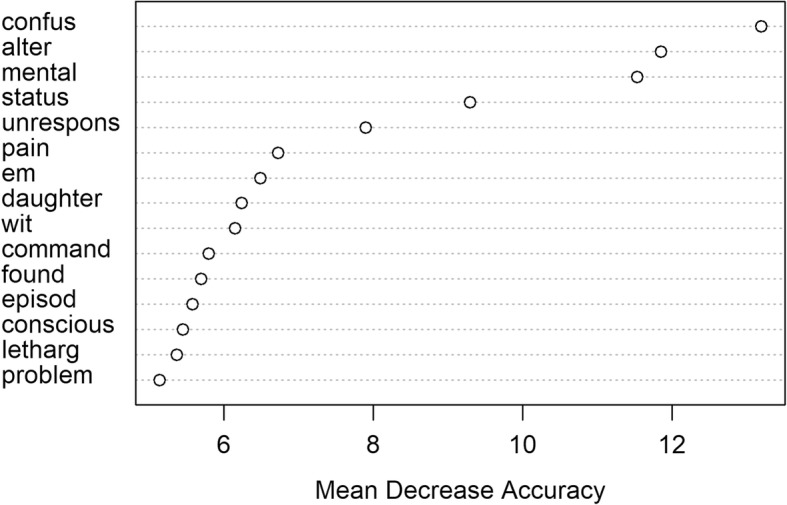
Variable importance plot based on the RF classifier

The accuracies of all the baseline BOW-based machine learning models exceeded the accuracy calculated based on ICD code designations. The best performing classifier within the BOW-based ones was the RF classifier with an accuracy of 92.1% and AUC of 97.5% (Table 4 and Fig. 2). However, the Lasso and SVM were almost as good with AUC’s of 97.3 and 96.7% respectively. We also examined variable importance from the RF model, which provides insight on significant words (Fig. 3). Note that the words are stemmed: so “confus” may stand for “confusion” and “em” for “EMS” as in emergency medical services.

**Table 4:** Accuracy and area under the ROC curve (AUC) results for bag of words (BOW)-based models and the word embedding-based deep learning models along with 95% confidence intervals (CI)

Category	Model^a^	AUC (95% CI)	Accuracy	Epochs
BOW models	RF	0.975 (0.967–0.983)	0.921	N/A
	LASS	0.973 (0.964–0.982)	0.912	N/A
	SVM	0.967 (0.957–0.976)	0.912	N/A
	MLP	0.947 (0.934–0.960)	0.883	N/A
	SDT	0.934 (0.918–0.950)	0.911	N/A
	NBC	0.924 (0.908–0.940)	0.838	N/A
Deep learning models	CNN_D200	**0.985** (0.979–0.992)	**0.945**	30.8
	CNN_W2V	**0.985** (0.979–0.991)	0.942	**25.0**
	CNN_D50	0.984 (0.978–0.991)	0.944	36.6

^a^Model abbreviations are described in the text

The number of epochs for training the deep learning is based on the early stopping condition as described in the methods. The entries are sorted in descending order of AUC within each category. Bolding indicates results for the best performing models

### Word embeddings

The W2V model successfully clustered words that seemed to have similar semantic contexts (Additional file 1: Figure S1), for example the cosine similarity for “male” and “female” word embedding vectors was 0.99; “altered” and “mental” was 0.98; and “syncope” and “palpitations” was 0.98.

### Deep learning classifiers

The deep learning classifiers outperformed the BOW classifiers. The best accuracy overall was for the CNN_D200 (the CNN model with the untrained word embeddings and with embedding vector dimension of 200) and best overall AUC was a tie between the CNN_D200 and CNN_W2V (the CNN model with the word embeddings initialized by the pre-trained W2V model), although the differences are not significant between any of the CNN models. Nevertheless, all CNN models outperformed all the BOW models.

As implied above, pre-training the word embeddings using W2V, results in a similar performance as the other CNN models (Fig. 2b) with the advantage of a semantically meaningful word space represented through t-SNE (Additional file 1: Figure S1). Additionally, using the W2V to initialize the word embedding weights reduced the number of epochs needed to reach validation loss minima from an average of above 30 to around 25 epochs per k-fold training cycle (Table 4).

## Discussion

Our results showing relatively low accuracy of the ICD codes in identifying AMS symptoms in the ED visit setting are consistent with reports in the literature about ICD coding problems [19, 20]. The BOW-based classifiers are significantly more adept than ICD codes at the automated identification of these symptoms within the clinical notes. Amongst this category of classifiers, the most notable performance was seen in the RF and Lasso models. However, the word embedding deep learning models resulted in an even higher accuracy and AUC. Intuitively, this is not surprising given that the word embedding based models preserve the order of words and can “learn” from the patterns derived from word sequences. This is in contrast to the classical BOW-based models, which when used as unigrams depend simply on individual word frequencies, albeit normalized, as features. It should be noted that the best performing classifiers (from both categories) are reaching performance levels that are close to ceiling for this classification task, which bodes well for pulling actionable information from the clinical notes for patients at risk of complications following a pulmonary embolism. However, the close to ceiling results, especially in the deep learning models, make it more difficult to compare performances across models. This may be due to the fact that this is a very focused binary classification task to identify a cluster of symptoms related to AMS in one type of clinical notes, namely HPI’s.

One advantage for the classical models such as decision trees and RF is ease of interpretability. Our variable importance analysis based on the RF model (Fig. 3) provides insight into the strong performance on this supervised learning task. This analysis confirms our intuition about the significance of words such as “confusion” and “altered” as key words in identifying the AMS cluster of symptoms in the context of pulmonary embolism, which are also the symptoms that our clinical team looked for during the labeling of the data. Some words with high importance (or low mean decrease in accuracy), such as “em” for EMS and “daughter”, are more difficult to explain, but could hint to a higher probability of AMS if a patient were brought to the ED by EMS or accompanied by a caring family member. In fact, these results suggest that it may be beneficial to use both types of models when addressing a given problem, leveraging advantages of deep learning models, as well as advantages of interpretable models.

Table 4 shows that deep learning models perform fairly well on this supervised machine learning binary classification task for identifying a single symptom or a cluster of symptoms such as AMS; however, these models could easily be expanded to support more complex multi-class, multi-label tasks using deep learning neural networks such as the ones used in image annotation experiments [53].

As noted in the results section, the W2V model was successful in pre-learning word similarities, from the unlabeled ED notes and provided a semantically meaningful representation with clusters of high salience words (i.e., altered, mental, status: see Additional file 1: Figure S1). The use of pre-trained W2V models to initialize the weights in the word-embedding layer results in fewer epochs during training likely due to faster convergence during the gradient descent algorithm. Moreover, the CNN with W2V embeddings had AUC performance levels well within the 95% confidence intervals of the other CNNs. On examining the ROC curve in Fig. 2b, it is difficult to discern the advantage of one CNN model over any of the others used in this study. Aside from faster training and the lack of reliance on labor-intensive labeling, more data is likely needed to ascertain the advantage on performance, or lack thereof, of pre-training with word2vec.

On examining some of the misclassified text notes, we identified a few false positives due to distant negations: for example: “primary symptoms do not include headaches, syncope” or “patient denies fevers, chills, confusion”. This could be improved by including negation detection [54] in our pre-processing of the text. Another approach that has been used for improving performance on complex classification tasks is the application of an attention mechanism to the neural network [55], which has been used effectively in the classification of radiology reports with the added benefit of interpretability and demonstration of text feature salience [56]. Finally, the good performance demonstrated by many of the models here, in particular the CNN models, is a promising outcome in favor of automating tasks such as chart reviews, which are often perceived as expensive and time consuming when performed by a human. In contrast, it takes a trained model a fraction of a second to classify clinical text, an HPI document in this case, with reliable results that may be useful down the line for consumption by a clinical decision support tool.

### Limitations and future directions

This study draws on data from one EHR system at a single academic medical center making it difficult to draw generalizations about the high level of performance of the CNN-based models in other environments. The performance of these models, as well as the classical machine learning algorithms, was only examined through the narrow prism of a simple text classifier to identify AMS in one type of EHR clinical text, namely the HPI. Future work should include collaboration with other institutions to ascertain the performance of these models in other environments, as well as the examination of other types of clinical notes (e.g. physical exam notes) and the broader applications of machine learning tasks in decision support and health outcomes.

## Conclusions

Several of the machine learning models described above, performed fairly well on these focused symptom detection tasks in clinical text. These include the traditional BOW-based RF model. However, all the deep learning models based on the convolutional neural architecture presented here outperformed all the classic BOW-based models. The application of pre-training on a large unlabeled text corpus with an algorithm such as word2vec may hasten the training during the supervised learning process, which could be advantageous with larger data sets.

The high levels of performance in these models bode well for risk modeling and actionable decision making. The results seem promising for the eventual use of these types of classifiers as a component in support of clinical decision support, especially when combined with other sources of information from the EHR. The ultimate goal is the improvement, increased comprehensiveness and reliability of information used for applications in risk stratification tools such as PESI for pulmonary embolism.

## Additional file

Additional file 1: **Figure S1.** Two dimension t-SNE mapping of the word2vec model word vectors showing a subset of the vocabulary. (PDF 14 kb)

## Data Availability

The data set cannot be shared publicly in order to protect the confidentiality and privacy of individuals, since the clinical text notes could not be completely and conclusively scrubbed from personal identifiers. Data are available from the Medical University of South Carolina Data Review Committee and Office of Research Integrity (contact via the author: Dr. Obeid) for researchers who meet the criteria for access to confidential data and who have appropriate Ethics or Institutional Review Board approval for human research.

## Abbreviations

Adam: Adaptive moment estimation gradient
AMS: Altered mental status
AUC: Area under the curve
BOW: Bag-of-words
CI: Confidence interval
CNN: Convolutional neural networks
D200: Word embedding with 200 dimensions per word
D50: Word embedding with 50 dimensions per word
ED: Emergency department
EHR: Electronic health records
HPI: history of present illness
ICD: International Classification of Diseases
IRB: Institutional Review Board
LASS: Lasso
MUSC: Medical University of South Carolina
NBC: Naïve Bayes classifier
NLP: Natural language processing
PESI: Pulmonary Embolism Severity Index
RDW: Research data warehouse
REDCap: Research Electronic Data Capture
ReLU: Rectified linear unit
RF: Random forest
ROC: Receiver operating characteristic
SDT: Single decision tree
SVM: Support vector machines
tf-idf: Term frequency–inverse document frequency
t-SNE: t-distributed stochastic neighbor embedding
UMLS: Unified Medical Language System
W2V: word2vec
WE: Word embeddings
