# Supplementary figures and images for: Automated detection of altered mental status in emergency department clinical notes: a deep learning approach
Source: BMC Med Inform Decis Mak. 2019 Aug 19;19:164. doi: 10.1186/s12911-019-0894-9 (PMC6701023; doi:10.1186/s12911-019-0894-9)

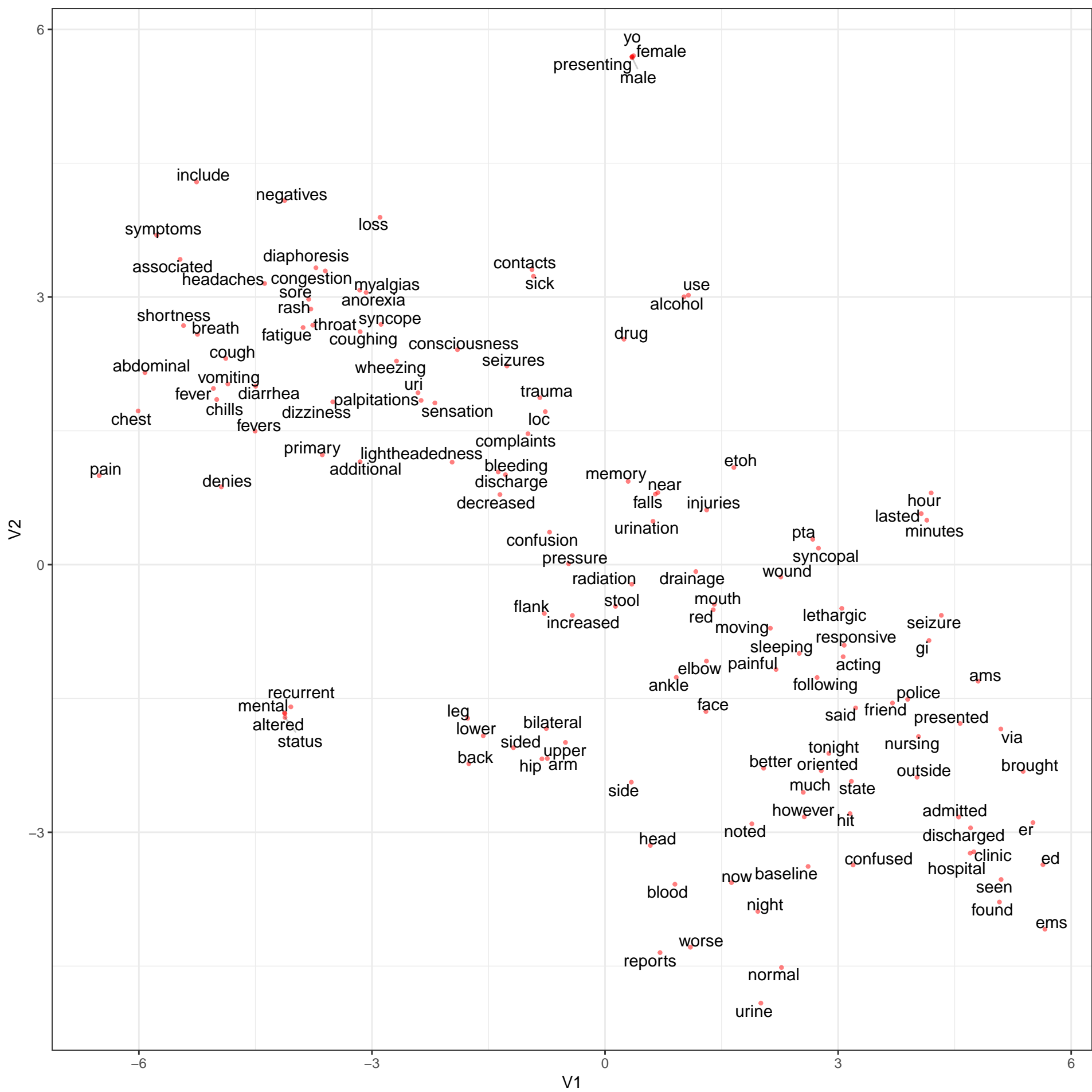

Supplement: Supplementary file 1 — Figure S1. Two dimension t-SNE mapping of the word2vec model word vectors showing a subset of the vocabulary. (PDF 14 kb) [file 12911_2019_894_MOESM1_ESM.pdf]
